# Supplementary material for: Objective evaluation of visual fatigue in patients with intermittent exotropia
Source: PLoS One. 2020 Mar 26;15(3):e0230788. doi: 10.1371/journal.pone.0230788 (PMC7098610; doi:10.1371/journal.pone.0230788)
Supplement: S1 Fig — Questions 1–3 were designed to assess subjective eye symptoms and Questions 4–7 to assess physical and mental discomfort. The total scores for Q1–3 were used to assess visual fatigue resulting from the visual task. n.p.: no problem. (DOCX) [file pone.0230788.s001.docx]

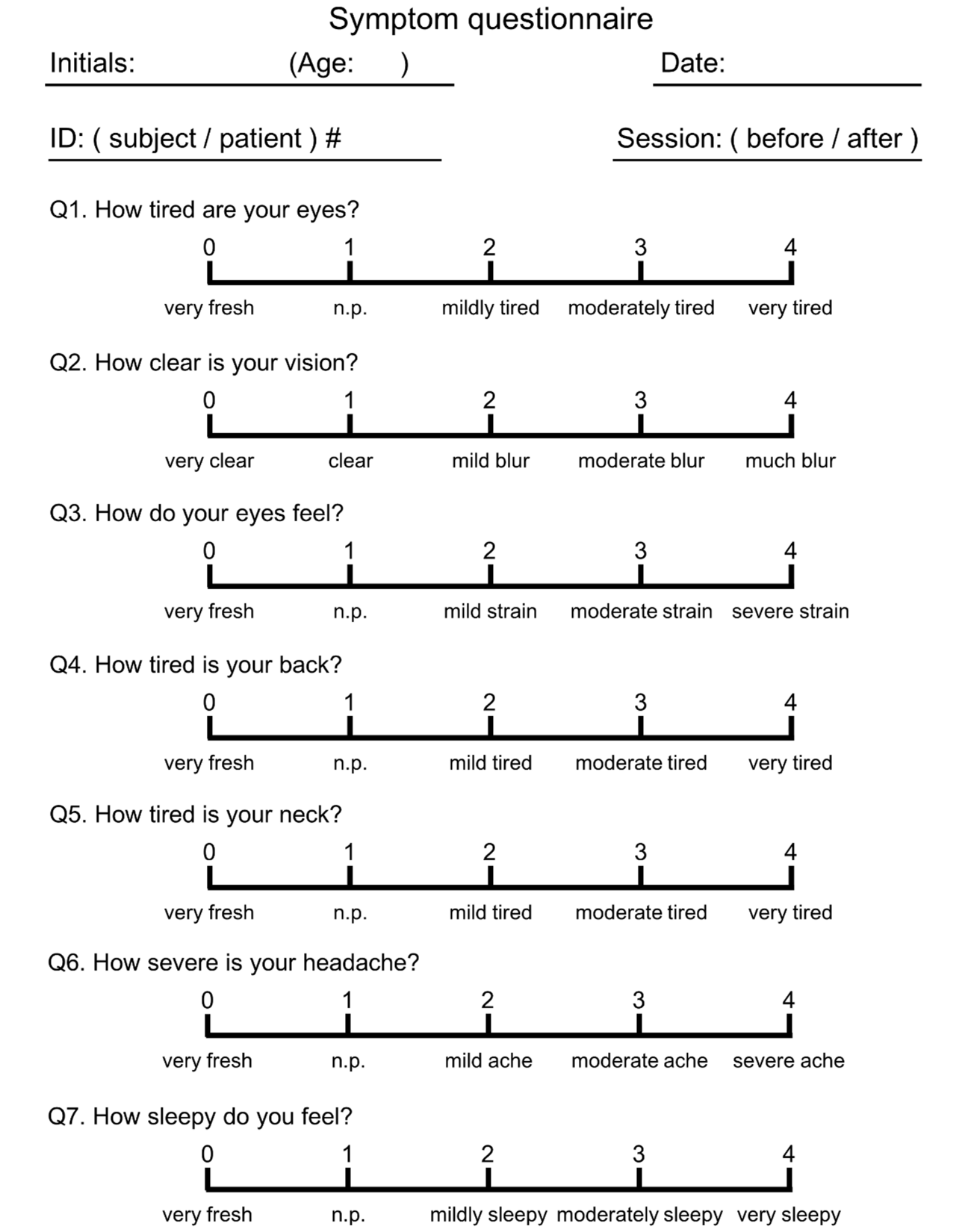


**Supplemental Figure 1. The subjective symptom questionnaire**

Questions 1–3 were designed to assess subjective eye symptoms and Questions 4–7 to assess physical and mental discomfort. The total scores for Q1–3 were used to assess visual fatigue resulting from the visual task.

n.p.: no problem
