# Supplementary material for: Objective evaluation of visual fatigue in patients with intermittent exotropia
Source: PLoS One. 2020 Mar 26;15(3):e0230788. doi: 10.1371/journal.pone.0230788 (PMC7098610; doi:10.1371/journal.pone.0230788)
Supplement: S1 Table — The error term is the standard deviation. The normality of previsual task were analyzed by the Shapiro-Wilk test. BFM, binocular fusion maintenance; NPC, near point of convergence; PD, prism diopter. (DOCX) [file pone.0230788.s003.docx]

**Supplementary Table 1. Distribution for the intermittent exotropia (IXT) group in the previsual task**

|  |  | Shapiro-Wilk test | | | |  |
| --- | --- | --- | --- | --- | --- | --- |
| Test | Previsual task | *W* value | | *P* value | |  |
| BFM | 0.915 ± 0.119 | | 0.710 | | <0.001 | |
| NPC (cm) | 4.7 ± 3.2 | | 0.684 | | <0.001 | |
| Fusional vergence range (PD) | 23.6 ± 8.7 | | 0.880 | | 0.048 | |
| Subjective symptom questionnaire |  | |  | |  | |
| Q1 | 207 ± 0.83 | | 0.868 | | 0.032 | |
| Q2 | 1.14 ± 0.53 | | 0.734 | | <0.001 | |
| Q3 | 2.00 ± 0.88 | | 0.837 | | 0.012 | |
| Q4 | 2.28 ± 1.14 | | 0.898 | | 0.088 | |
| Q5 | 2.21 ± 1.31 | | 0.911 | | 0.141 | |
| Q6 | 1.21 ± 1.12 | | 0.839 | | 0.013 | |
| Q7 | 1.43 ± 1.01 | | 0.887 | | 0.061 | |

The error term is the standard deviation. The normality of previsual task were analyzed by the Shapiro-Wilk test. BFM, binocular fusion maintenance; NPC, near point of convergence; PD, prism diopter.

**Supplementary Table 2. Distribution for the intermittent exotropia (IXT) group in the postvisual task**

|  |  | |  | | | |
| --- | --- | --- | --- | --- | --- | --- |
| Test | Postvisual task | | W value | | *P* value | |
| BFM | 0.729 ± 0.252 | 0.877 | | 0.043 | |  |
| NPC (cm) | 5.6 ± 3.8 | 0.539 | | <0.001 | |  |
| Fusional vergence range (PD) | 21.1 ± 10.8 | 0.984 | | 0.991 | |  |
| Subjective symptom questionnaire |  |  | |  | |  |
| Q1 | 2.93 ± 0.83 | 0.859 | | 0.024 | |  |
| Q2 | 1.64 ± 1.01 | 0.880 | | 0.048 | |  |
| Q3 | 2.92 ± 0.92 | 0.881 | | 0.049 | |  |
| Q4 | 2.28 ± 1.07 | 0.880 | | 0.048 | |  |
| Q5 | 2.21 ± 1.12 | 0.881 | | 0.049 | |  |
| Q6 | 1.00 ± 0.96 | 0.844 | | 0.014 | |  |
| Q7 | 1.78 ± 1.25 | 0.881 | | 0.049 | |  |

The error term is the standard deviation. The normality of postvisual task were analyzed by the Shapiro-Wilk test. BFM, binocular fusion maintenance; NPC, near point of convergence; PD, prism diopter.

**Supplementary Table 3. Distribution for the control group in the previsual task**

| Test | Previsual task | *W* value | | *P* value | |  |
| --- | --- | --- | --- | --- | --- | --- |
| BFM | 0.947 ± 0.068 | | 0.759 | | 0.002 | |
| NPC (cm) | 1.8 ± 1.8 | | 0.518 | | <0.001 | |
| Fusional vergence range (PD) | 32.5 ± 8.9 | | 0.928 | | 0.26 | |
| Subjective symptom questionnaire |  | |  | |  | |
| Q1 | 1.00 ± 0.76 | | 0.754 | | 0.001 | |
| Q2 | 0.87 ± 0.64 | | 0.790 | | 0.003 | |
| Q3 | 1.13 ± 0.74 | | 0.816 | | 0.007 | |
| Q4 | 1.07 ± 0.79 | | 0.816 | | 0.007 | |
| Q5 | 1.00 ± 0.76 | | 0.822 | | 0.008 | |
| Q6 | 0.67 ± 0.62 | | 0.766 | | 0.002 | |
| Q7 | 1.00 ± 0.65 | | 0.586 | | <0.001 | |

The error term is the standard deviation. The normality of postvisual task were analyzed by the Shapiro-Wilk test. BFM, binocular fusion maintenance; NPC, near point of convergence; PD, prism diopter.

**Supplementary Table 4. Distribution for the control group in the postvisual task**

| Test | Postvisual task | *W* value | | *P* value | |  |
| --- | --- | --- | --- | --- | --- | --- |
| BFM | 0.917 ± 0.082 | | 0.839 | | 0.013 | |
| NPC (cm) | 2.6 ± 2.1 | | 0.779 | | 0.002 | |
| Fusional vergence range (PD) | 33.4 ± 7.6 | | 0.949 | | 0.52 | |
| Subjective symptom questionnaire |  | |  | |  | |
| Q1 | 1.67 ± 0.72 | | 0.782 | | 0.003 | |
| Q2 | 0.80 ± 0.41 | | 0.499 | | <0.001 | |
| Q3 | 1.46 ± 0.64 | | 0.743 | | 0.001 | |
| Q4 | 1.20 ± 0.86 | | 0.881 | | 0.050 | |
| Q5 | 1.07 ± 0.79 | | 0.816 | | 0.006 | |
| Q6 | 0.67 ± 0.48 | | 0.603 | | <0.001 | |
| Q7 | 1.28 ± 1.08 | | 0.884 | | 0.056 | |

The error term is the standard deviation. The normality of postvisual task were analyzed by the Shapiro-Wilk test. BFM, binocular fusion maintenance; NPC, near point of convergence; PD, prism diopter.
