# Supplementary material for: Objective evaluation of visual fatigue in patients with intermittent exotropia
Source: PLoS One. 2020 Mar 26;15(3):e0230788. doi: 10.1371/journal.pone.0230788 (PMC7098610; doi:10.1371/journal.pone.0230788)
Supplement: S2 Table — The error term is the standard deviation. The normality of postvisual task were analyzed by the Shapiro-Wilk test. BFM, binocular fusion maintenance; NPC, near point of convergence; PD, prism diopter. (DOCX) [file pone.0230788.s004.docx]

**Supplementary Table 2. Distribution for the intermittent exotropia (IXT) group in the postvisual task**

|  |  | |  | | | |
| --- | --- | --- | --- | --- | --- | --- |
| Test | Postvisual task | | W value | | *P* value | |
| BFM | 0.729 ± 0.252 | 0.877 | | 0.043 | |  |
| NPC (cm) | 5.6 ± 3.8 | 0.539 | | <0.001 | |  |
| Fusional vergence range (PD) | 21.1 ± 10.8 | 0.984 | | 0.991 | |  |
| Subjective symptom questionnaire |  |  | |  | |  |
| Q1 | 2.93 ± 0.83 | 0.859 | | 0.024 | |  |
| Q2 | 1.64 ± 1.01 | 0.880 | | 0.048 | |  |
| Q3 | 2.92 ± 0.92 | 0.881 | | 0.049 | |  |
| Q4 | 2.28 ± 1.07 | 0.880 | | 0.048 | |  |
| Q5 | 2.21 ± 1.12 | 0.881 | | 0.049 | |  |
| Q6 | 1.00 ± 0.96 | 0.844 | | 0.014 | |  |
| Q7 | 1.78 ± 1.25 | 0.881 | | 0.049 | |  |

The error term is the standard deviation. The normality of postvisual task were analyzed by the Shapiro-Wilk test. BFM, binocular fusion maintenance; NPC, near point of convergence; PD, prism diopter.
