# Supplementary material for: Objective evaluation of visual fatigue in patients with intermittent exotropia
Source: PLoS One. 2020 Mar 26;15(3):e0230788. doi: 10.1371/journal.pone.0230788 (PMC7098610; doi:10.1371/journal.pone.0230788)
Supplement: S3 Table — The error term is the standard deviation. The normality of postvisual task were analyzed by the Shapiro-Wilk test. BFM, binocular fusion maintenance; NPC, near point of convergence; PD, prism diopter. (DOCX) [file pone.0230788.s005.docx]

**Supplementary Table 3. Distribution for the control group in the previsual task**

| Test | Previsual task | *W* value | | *P* value | |  |
| --- | --- | --- | --- | --- | --- | --- |
| BFM | 0.947 ± 0.068 | | 0.759 | | 0.002 | |
| NPC (cm) | 1.8 ± 1.8 | | 0.518 | | <0.001 | |
| Fusional vergence range (PD) | 32.5 ± 8.9 | | 0.928 | | 0.26 | |
| Subjective symptom questionnaire |  | |  | |  | |
| Q1 | 1.00 ± 0.76 | | 0.754 | | 0.001 | |
| Q2 | 0.87 ± 0.64 | | 0.790 | | 0.003 | |
| Q3 | 1.13 ± 0.74 | | 0.816 | | 0.007 | |
| Q4 | 1.07 ± 0.79 | | 0.816 | | 0.007 | |
| Q5 | 1.00 ± 0.76 | | 0.822 | | 0.008 | |
| Q6 | 0.67 ± 0.62 | | 0.766 | | 0.002 | |
| Q7 | 1.00 ± 0.65 | | 0.586 | | <0.001 | |

The error term is the standard deviation. The normality of postvisual task were analyzed by the Shapiro-Wilk test. BFM, binocular fusion maintenance; NPC, near point of convergence; PD, prism diopter.
