# Supplementary material for: Objective evaluation of visual fatigue in patients with intermittent exotropia
Source: PLoS One. 2020 Mar 26;15(3):e0230788. doi: 10.1371/journal.pone.0230788 (PMC7098610; doi:10.1371/journal.pone.0230788)
Supplement: S4 Table — The error term is the standard deviation. The normality of postvisual task were analyzed by the Shapiro-Wilk test. BFM, binocular fusion maintenance; NPC, near point of convergence; PD, prism diopter. (DOCX) [file pone.0230788.s006.docx]

**Supplementary Table 4. Distribution for the control group in the postvisual task**

| Test | Postvisual task | *W* value | | *P* value | |  |
| --- | --- | --- | --- | --- | --- | --- |
| BFM | 0.917 ± 0.082 | | 0.839 | | 0.013 | |
| NPC (cm) | 2.6 ± 2.1 | | 0.779 | | 0.002 | |
| Fusional vergence range (PD) | 33.4 ± 7.6 | | 0.949 | | 0.52 | |
| Subjective symptom questionnaire |  | |  | |  | |
| Q1 | 1.67 ± 0.72 | | 0.782 | | 0.003 | |
| Q2 | 0.80 ± 0.41 | | 0.499 | | <0.001 | |
| Q3 | 1.46 ± 0.64 | | 0.743 | | 0.001 | |
| Q4 | 1.20 ± 0.86 | | 0.881 | | 0.050 | |
| Q5 | 1.07 ± 0.79 | | 0.816 | | 0.006 | |
| Q6 | 0.67 ± 0.48 | | 0.603 | | <0.001 | |
| Q7 | 1.28 ± 1.08 | | 0.884 | | 0.056 | |

The error term is the standard deviation. The normality of postvisual task were analyzed by the Shapiro-Wilk test. BFM, binocular fusion maintenance; NPC, near point of convergence; PD, prism diopter.
