# Supplementary material for: Objective evaluation of visual fatigue in patients with intermittent exotropia
Source: PLoS One. 2020 Mar 26;15(3):e0230788. doi: 10.1371/journal.pone.0230788 (PMC7098610; doi:10.1371/journal.pone.0230788)
Supplement: Attachment — Submitted filename: Hirota_PlosOne_Response_to_reviewers_rev2.docx [file pone.0230788.s008.docx]

Reviewer #1: The authors answer all my questions. By the way, I couldn't find the Movie 1 in this version either.

- Thank you for the re-review. My colleagues and I were able to improve the manuscript thanks to your constructive comments.
- I uploaded a movie file in the file inventory at the time of the first revision (Photo 1). I have carefully confirmed that the movie is visible. You can see the video by clicking the pdf of Movie1.

Reviewer #2: Thank you for taking the time to address the previous comments. However, I still have some concerns, especially regarding the methodology, that need addressing.
The line numbers refer to the track changes version document.
The procedure of measuring BFM is not clear. Two new sentences have been added from line 116. The second sentence would be better placed at the end of that p/g, on line 127. The second sentence is also unclear: ‘fusion break can be judged automatically to record the eye movements because one eye deviates’. The underlined text does not make sense. Is the fusion break determined by the equipment or by the examiner viewing the eyes, looking for one eye to deviate?
In my previous comments I had asked what liquid crystal shutters are but this has not been addressed. Please explain what these are for? Are they used to reduce light intensity?

- Thank you for your constructive comments. We have revised our manuscript in accordance to your comments. The line numbers refer to the track changes version document.
- The second sentence has been moved from line 133 to line 135. We have used the word “automatically” to mean objectively evaluating with the BWFA.
- We would like to apologize for not providing an explanation. We used liquid crystal shutters to reduce light intensity (line 137 – 138).

145 Is this target viewed on the plate or a screen?

- We used a printed plate and not a digital screen (line 158).

151 Remove ‘changed just in’ as the last part of the sentence does not follow with this included. It is better without.

- We have removed the words “changed just in” (line 166).

149-58 It is not clear what target the subjects are looking at during this procedure. Is it the same one that was used to correct refractive errors.
It is still not clearly stated what you are measuring in the BFM test. Is it the intensity of light when then fusion breaks? What are the units? This is important to include and could be added on line 157.

- We have modified the sentences to explain the procedure.
- The fixation target used was same as that the one used to correct refractive errors (lines 162 – 163).
- The BFM test evaluated the intensity of incident light ratio with both eyes when binocular fusion breaks (lines 175–176).

184 How was the target moved back and forth? Was this on a motorized beam?

- We moved the target using an electric motor (line 202).

189 All subjects underwent four trials – what is a trial? Do you mean 4 x the visual task? The motion was performed 3 times, so in total 12 motions, before all the measurements were repeated?

- We defined the 1 trial as three reciprocating motions (lines 202–203). The subjects underwent 4 trials, so 12 motions in total.

189 Make it clearer that the eye movements and accommodation were measured during the visual task.

- We have modified the sentences; eye movements and accommodative responses were measured simultaneously during the visual task.

190 Accommodation has not been previously mentioned. A response to my previous comment explains the wavefront aberrometer measures this. This needs to be included earlier on 149 where it states what measurements are taken.

- We have mentioned that we measured the wavefront aberrations of second orders (accommodative response) (line 164).

199 Unclear justification provided to previous comment on why Q4-7 need to be included. These are not significant anyway. If keeping them in, then you should state somewhere what the questions were. Q1-3 have now been included in the text so the same could be done with these. Alternatively, include the questions in a table.

- We apologize for the misunderstanding. We used Questions 4–7 to avoid bias. As you pointed out, Questions 4–7 are less important than Questions 1–3. We described the contents of Questions 4–7 in the main text (lines 224–233).

291 Would it be better to present the healthy volunteer results first, in figure 1? And the IXT results in figure 2?
In my previous comments I had asked how you determined if binocular coordination had been disrupted. This was answered in the response document but has not been included in the manuscript. It would be helpful to the reader, as it was assumed this was done by eyeballing the data.

- We have changed the figure number of 1 and 2. The healthy volunteer results are shown in Figure 1, whereas IXT results have been shown in Figure 2.
- We have mentioned about checking of binocular coordination from the numerical data and anterior video data (lined 357–358).

Figure 4 and 5 legends still have results included. Results should be included in the main manuscript and not in the legend.

- The data of quartiles, medians, and 95% confidence intervals have neem moved from the legends to the main text.

Table 4. You appeared to have misread by previous comment. I had asked if NPC and fusional vergence was also change in values from pre- to post- task, like the BFM and survey. You had answered yes but did not change the table accordingly. I would suggest changing ‘Test’ to ‘Change in test’ or ‘change in test result’? Th at way it is clear that all the tests listed are looking at the change. Remove ‘change’ in front of BFM and subjective symptom questionnaire as the new heading now makes this clear.

- We have misread your comment in the first revision. The subgroup in IXT did not find significantly differ in other parameters. We have changed the table number from 4 to 5 and have also changed the columns from “Test” to “Change in test.”

422 It is not clear how or why Q4-7 would related to BFM, NPC scores etc.

- We added the analysis for total subjective physical and psychological discomfort (line 304 – 305, 309 – 310, 513 – 514, 537 – 548).

432 It is stated that in a previous study BFM was significantly decreased but it is not stated who was being tested. What condition did they have or were they controls?

- We mentioned that the previous study evaluated the BFM in healthy volunteers (line 640).

435 Consider the structure. Already covered survey questions in an earlier p/g, then moved onto BFM, and now coming back to survey questions. The next p/g moves onto discussing BFM again.

- We have modified the structure. This paragraph has been merged with the fourth paragraph of the Discussion.

467 You’ve now made it clear that the change is related to after performing the visual task but this information is not presented in the correct part of the sentence. ‘The change after a visual task in BFM was significantly lower’ should actually be ‘The change in BFM, after a visual task, was significantly lower’. This should be changed in the next sentence too.

- We have revised the sentence as per your comments (lines 722–724).

473 I had commented that ‘at the zero point’ was not clear. This has been changed to ‘none’ but the sentence still does not make sense. I don’t understand what point is being made. Revise this sentence.

- The phrase “At the zero point” means “the subjects were not aware of visual fatigue.” We have mentioned this in the Conclusion section (lines 727 – 728).

Carefully proof read the manuscript as there are still many typographical and grammatical errors. Here are some of them:

- We have carefully proofread our manuscript. Furthermore, the manuscript has been proofread by native English speakers.

- 74 ‘to align both eyes for obtain’ should be ‘to align both eyes to obtain’

- We have revised the word “for obtain" to “to obtain” (line 70).

- 140 ‘eye movements used to calculate’ should be ‘eye movements were used to calculate’

- We have added the word “were” (line153).

- 231 avoid ‘we’/first person

- We have avoided the use of first person (lines 276).

- 298 ‘showed similar to the representative data’. Do you mean showed similar results?

- Yes, it does. All healthy volunteers maintained binocular coordination during the visual task. In all patients with IXT, binocular coordination was gradually disrupted during the visual task (lines 368 – 371).

- 398 ‘vergence and accommodation working simultaneously’. Do you mean ‘vergence and accommodation were recorded simultaneously’?

- Yes, it does. I have revised “working” to “recorded” (line 599).

- 420 ‘at the starting visual task’ needs re-writing

- As per your suggestion, we have rewritten the sentence (lines 614 – 616).

- 450 ‘did not significantly different’ should be ‘did not significantly differ’.

- We have revised the word “different” to “differ” (line 686).
